# (Dis)agreement on Sight-Singing Assessment of Undergraduate Musicians

**DOI:** 10.3389/fpsyg.2018.00837

**Published:** 2018-05-29

**Authors:** Graziela Bortz, Nayana G. Germano, Hugo Cogo-Moreira

**Affiliations:** ^1^Department of Music, Universidade Estadual Paulista Júlio de Mesquita Filho, São Paulo, Brazil; ^2^Department of Psychiatry, Federal University of São Paulo, São Paulo, Brazil

**Keywords:** sight-singing assessment, inter-judge validity and reliability, music performance assessment, conservatoire training, music evaluation

## Abstract

Assessment criteria for sight-singing abilities are similar to those used to judge music performances across music school programs. However, little evidence of agreement among judges has been provided in the literature. Fifty out of 152 participants were randomly selected and blindly assessed by three judges, who evaluated students based on given criteria. Participants were recorded while sight-singing 19 intervals and 10 tonal melodies. Interjudge agreement on melodic sight-singing was tested considering four items in a five-point Likert scale format as follows: (1) Intonation and pitch accuracy; (2) Tonal sense and memory; (3) Rhythmic precision, regularity of pulse and subdivisions; (4) Fluency and music direction. Intervals were scored considering a 3-point Likert scale. Agreement was conducted using weighted kappa. For melodic sight-singing considering the ten tonal melodies, on average, the weighted kappa (κ_w_) were: κ1_w_ = 0.296, κ2_w_ = 0.487, κ3_w_ = 0.224, and κ4_w_ = 0.244, ranging from fair to moderate.. For intervals, the lowest agreement was kappa = 0.406 and the highest was kappa = 0.792 (on average, kappa = 0.637). These findings light up the discussion on the validity and reliability of models that have been taken for granted in assessing music performance in auditions and contests, and illustrate the need to better discuss evaluation criteria.

## Introduction

### Sight-Reading and Sight-Singing Skills

Sight-reading is a required skill for all students in undergraduate and graduate music programs; it is needed for activities such as piano accompaniment, chamber music, and various other ensemble practices. Some of the tools that are conceived to assess music performance actually address sight-reading ability, including the Watkins-Farnum Performance Scale (Watkins and Farnum, 1954), which consists of 14 exercises of increasing difficulty. They are dichotomous items, as performances are assessed in terms of right or wrong: “Wrong notes, rhythms and articulations are considered errors, as is failure to observe a dynamic marking. Poor tone quality or intonation is not considered an error" (Haley, 1999, p. 169). The Watkins-Farnum Performance Scale has been used in placement auditions for band students in elementary and high schools in the United States, and it has been criticized for being restricted primarily to the sight-reading aspect of music.

In contrast to the naturalistic assessment paradigm cited above, computer-based tools can be used to evaluate sight-reading; for instance, Kopiez et al. (2006), tested 52 piano students who used a MIDI piano to accompany a prerecorded violin part played with a metronome. Kopiez et al. (2006) used a program called Midi Compare (Dixon, 2002) to match the score with the pitches that the participants recorded while sight-reading an accompaniment. Although this is an automatized process, as it does not consider agogic music or musicians’ live interactions (even considering an adjustable critical time frame with a margin of 0.25 s), Kopiez et al. (2006) found that there is a correlation between performance skills (speed of trills and wrist tapping) and the speed of information processing. Their findings led Kopiez et al. (2006, p. 23) to the conclusion that “sight-reading achievement, at a very high level in expert pianists, is determined by acquired expertise as well as by other factors such as ‘speed of information processing’ and ‘psychomotor speed.’

Sight-reading and sight-singing terms have been used interchangeably in music. The former refers mainly to the practice of reading music without previous preparation or study with the instrument; the latter means the same but for the voice instead of an instrument. Sight-singing is part of ear-training programs for both instrumentalists and singers in music schools, in which case the aural construction has to be done while reading the music notation, without the help of an instrument. These skills are expected to be correlated; however, the way they are captured is distinct. In this paper, we specifically address the evaluation of sight-singing skills.

### Sight-Singing Assessment

Karpinsky (2000, p. 191–193) discussed the transferability of sight-singing to instrumental and vocal performance; although scores can be easily ascribed for categorical tests of music dictation and sight-reading using instruments (in terms of numbers of errors, as in the Watkins-Farnum Performance Scale; Watkins and Farnum, 1954), ascribing grades for other aspects of music making—such as phrasing, intonation, or rhythmic precision—is not an easy task. For example, Karpinsky discussed assessment tools and evaluation rubrics in Chapter 3, which is dedicated to melodic dictation, but he did not mention the issue when addressing sight-singing in Chapter 7.

The Associated Board of the Royal Schools of Music [ABRSM] (2017) presented rubrics and criteria for sight-reading and sight-singing; although those criteria are highly respected, Scott et al. (2016, p. 195) affirmed that “there are no technical reports available to support” the claim that they provide reliable tools for measurement. The Associated Board of the Royal Schools of Music [ABRSM] (2017) criteria included rhythmic accuracy; notes, pitch, and keys; accuracy; tempo; and continuity, which are also some of the most commonly used criteria to evaluate performance. ABRSM presented them in a broad way, as the items are apparently not evaluated categorically.

Because we are concerned not only with the assessment of sight-singing in terms of correct and incorrect pitches, keys, and rhythms, but also with the interpretational aspects of reading, we decided to examine the tools used to evaluate music performance.

### Music Performance Assessment

In 1987, Boyle and Radocy addressed the subject of accountability in musical performance, describing various ways through which it could be evaluated (Likert scales, paired comparisons, magnitude estimation, and rank orders). However, Boyle and Radocy (1987, p. 171) believed that, although only “certain aspects of music” could be measured, “the total musical experience based on the physical aspects remains subjective.”

One instance of such belief is that, despite the great impact biases have on judging musical performance, auditions for music jobs and competitions continue to happen without a screen. Even when auditions for orchestras are blind in the initial rounds, the semifinals and finals frequently occur openly.

The meta-analysis of Platz and Kopiez (2012, p. 75) showed “that the visual component is not a marginal phenomenon in music perception, but an important factor in the communication of meaning.” However, examples of biases in evaluating music performance can be witnessed in Tsay (2013), who described the great impact that visual cues have on sound when evaluating pianists in high-level solo competitions.

Goldin and Rouse (2000) conducted a study on sex-biased hiring in professional orchestras, comparing data before and after screens started to be used in auditions:

using the audition data, we find that the screen increases by 50 percent the probability that a woman will be advanced from certain preliminary rounds and increases by several fold the likelihood that a woman will be selected in the final round. (p. 738)

This, in turn, raises the issue about the need for discussing and carrying on further investigations that address the criteria that are used (or thought to be used) in music performance, as they affect the lives of many people.

### Assessment Instruments

In terms of instrumental performance, researchers have typically used one main indicator of global performance, such as Bergee (2007), who evaluated wind instruments in terms of scores from 0 (*poor*) to 100 (*excellent*). Thompson and Williamon (2003) evaluated the musical-instrument performance of college students using 13 categories and an overall rating using a 7-point Likert scale.

Hash (2012) proposed to evaluate both aspects: concert performance and sight-reading. Hash assessed the former using the following categories: tone, intonation, technique, balance, interpretation, musical effect, and other factors. For the sight-reading aspect, Hash’s categories were technical accuracy, flexibility, interpretation, musical effect, and general comments. For each category in Hash’s study, judges used a Roman-numeral scale from I (*superior*), to V (*poor*).

Wesolowski (2016) developed a tool to assess jazz big-band performance (the Jazz Big Band Performance Rating Scale); it constituted 18 items with a 4-point Likert-scale structure and was used to evaluate four domains (balance and blend, time-fell, idiomatic nuance, and expression). Zdzinski and Barnes (2002) assessed the string-instrument performance of high-school and middle-school students using 28 items on a 5-point Likert scale; the items were grouped into five factors: interpretation or musical effect, articulation or tone, intonation, rhythm or tempo, and vibrato.

Bergee (1993) evaluated randomly chosen brass students during jury performances and later replicated these evaluations for eight new, randomly chosen students. Agreement among judges under those circumstances might suggest viable items for composing a trait model, and the level of the interjudge reliability assessed via product-moment correlations ranged from 0.83 to 0.89 (Bergee, 1993, p. 20–22).

Examining the assessment rubric used in Kansas state high schools for large-group festivals, Latimer et al. (2010, p. 173) found that rhythm was unexpectedly “less reliable than other dimensions.” They mentioned another study (Norris and Borst, 2007) that reported similar findings.

Beliefs regarding subjectivity in the evaluation of art music are reflected in the fact that there are no valid and reliable scales for assessing the performance of musicians with high levels of expertise. Thompson and Williamon (2003) investigated the correlations in three judges’ evaluations of 61 performance students from the Royal College of Music in London; the performances were recorded using a digital video camera, according to the criteria of the ABRSM. Thompson and Williamon (2003) found only a moderate correlation among the evaluations.

Wrigley and Emmerson (2011) addressed the quality of music performance evaluations using a 7-point-Likert scale that assessed three domains: technical mastery and control, sound quality, and convincing musical understanding. Russell’s (2015) Aural Musical Performance Quality Measure used a Likert scale to evaluate solo-instrument musical performance; it comprised 44 items, divided into 11 subscales, and Russell found moderately consistent to consistent levels of reliability.

Jones (1986) examined singing assessments of 30 videotaped high school students. A Vocal Performance Rating Scale (VPRS) was built, and fifteen judges evaluated the videotapes considering 32 items (called factors) in three panels using a 5-point-Likert scale ranging from *strongly agree* to *strongly disagree*. Both aural and visual aspects of performance were investigated separately. Although judges reacted differently to visual aspects of performance, interjudge reliability reached 0.894, 0.917, and 0.920 for each group of the three panels, respectively.

### Reliability of Assessment

In statistical terms, regarding measures of interrater reliability, it is common to find works that use Spearman/Pearson correlations as measurements of agreement between continuous measures. However, such statistical inferential procedures are not adequate to such purposes, as both are indices of measurement for linear correlation between at least one scalar (continuous) measurement, thus conveying little useful information about the level of agreement; the statistical significance of the correlation is even less helpful (Altman and Gardner, 1988).

A review about statistical techniques used to measure agreement between continuous measures showed the correlation coefficient (aka Pearson correlation) to be commonly used for such aim (Zaki et al., 2012). However, the correlation coefficient (r) reflects the noises and direction of a linear relationship (Daly and Bourke, 2000), where perfect correlation between two continuous measures will be depicted if all the points lie along a straight line. If we compare two forms of evaluations (*Y* and *X*), it is possible to observe perfect correlation (*r* = 1) under the following two situations: *Y* = *X* or *Y* = 3*X*. Conversely, it is important to note that in terms of agreement, perfect agreement occurs only in the first situation (i.e., *Y* = *X*) but not in the latter, where obviously the value of Y is the triple of X (i.e., no agreement). In Zaki et al. (2012), the authors exemplified such a situation, demonstrating that the correlation coefficient r for the relationship between variables *A* and *B* is 0.9798. Even though the variable C is twice the value of B, the correlation coefficient of A and C is exactly the same (*r* = 0.9798). As a consequence, it is noticeable that there is no agreement between A and C, but the correlation coefficient value is still very high, suggesting a strong (linear) correlation or (linear) association. Consequently, the correlation coefficient does not represent agreement.

The Guidelines for Reporting Reliability and Agreement Studies (GRRAS) was proposed by Kottner et al. (2011), and, for agreement of continuous measures, the following techniques were cited: proportions of agreement (ranges), proportions of specific agreement (ranges), standard errors of measurement, coefficients of variation, Bland-Altman plots and limits of agreement. In terms of reliability, the statistical method is the intraclass correlation coefficient.

Finally, the aim of the present study is to test interjudge agreement for undergraduate music students participating in two tasks: melodic and interval sight-singing. Since criteria that will be examined here have been used in conservatories and music schools to evaluate those abilities, it is important to examine them in terms of validity and reliability. In order to investigate sight-singing assessment in terms of interjudge agreement, we examined the above available instruments used to evaluate music performance, sight-reading, and sight-singing, since those abilities share similar challenges, including visual and gender biases and criteria. Although many scales proposed to measure such abilities and interrater agreement are available, we found them problematic in terms of statistical reliability with consequences for both music students and professional assessment. Because we noticed the importance of visual biases in assessing music performance, we chose to conduct our study using audio recording exclusively, so we could observe if our musical criteria could be validated.

## Materials and Methods

### Methods

#### Participants and Study Design

Our eligible sample consisted of 152 undergraduate students (*n* = 92 male; 60.5%) from first to fourth year of study at *Instituto de Artes* (UNESP), randomly selected, who signed the consent form in accordance with the Declaration of Helsinki, and approved by *Plataforma Brasil*, no. 1.079.293, Ethics Committee of *Instituto de Ciência e Tecnologia Campus São José dos Campos*–UNESP Ethics Statement, to participate in broader research concerning the creation and validation of various tools to assess the music-perception skills of undergraduate music students. The participants’ mean age was 23.66 (*SD* = 5.5; range = 17–51), and they had an average of 10.57 years of musical practice (*SD* = 4.9). Because this study is focused exclusively on the evaluation of interjudge agreement, we selected a random subsample of 50 students to participate in the evaluation of interjudge validation. This random selection of 50 of the 152 students was implemented using atmospheric noise (a truly random algorithm; Haahr, 1998). Three specialists then assessed the 50 participants to test interjudgment agreement, finding that the participants did not differ statistically from the eligible sample of 152 students in terms of age, sex, or years of practice.

Participants received a numeric identifier (ID), and we audio-recorded them using a portable TASCAM recorder (model DR-05, configured in 16-bit Waveform Audio File Format; sample: 44.1 kHz, mono; low cut: 80 Hz).

For melodic sight-singing, we instructed the 152 participants that they would hear the tonic triad played on a Yamaha (U1 model) upright piano in a regular music classroom. In case of modulation, the triad played would be that of the initial tonic. In case they made an error, we also instructed them that they would have a second chance only if the whole melody had not already been sung. We announced the participants’ IDs at the beginning of each recording, followed by the numbers of each of the 10 melodies and the triad of each melody. We composed the melodies (Appendix A) in increasing order of difficulty for this test.

To address sight-singing abilities, we chose not to assess them in terms of number of errors; instead, we used four criteria (also called *items*, both in this the manuscript and in psychological assessments) that are commonly used in performance-evaluation tests and competitions: (a) pitch accuracy and intonation, (b) tonal sense and memory, (c) rhythmic and metric precision, and (d) fluency and direction. These are described in the Materials section and detailed in Appendix C.

The interval test took place on the same occasion, using the same sample of 50 randomized participants, who were advised that the first note of the music sheet’s written interval would be played. In some cases, the student sang the interval and quickly tried again; we instructed the judges not to consider the second attempt. We announced the participant ID at the beginning of each recording, followed by the number for each of the 19 intervals and the first note of each.

We later edited the recordings using a plug-in X-noise to filter out ambience noises such as air conditioning and to optimize the audio quality. We eliminated silence and pauses to facilitate the judges’ assessment.

#### Judges’s Selection Criteria

The three judges used the same four criteria (see section “Materials and Methods”) to assess the participants’ sight-singing skills for 10 tonal melodies. The participants’ names were blinded during the scoring procedures; judges only had access to the participants’ numeric IDs. The three judges independently assessed the 50 recordings, which they accessed via Dropbox. During the rating process, the judges were in separate, isolated rooms, and there was no communication among them. They had a deadline of 15 working days to finish all the ratings. Although the recordings were not randomized, the judges were blinded for all of them; they had access to the Dropbox recordings but had no specific instructions regarding the order in which to complete the assessment.

We selected judges based on the following criteria:

(1) They should not be singers. The participants were not all singers, as they included students of different major instruments, conducting, and music education. Thus, the level of vocal technique demanded should not be what would be expected from a singer.
(2) They should be tenured professors who are experienced in teaching within the music departments of *Instituto de Artes* (UNESP).
(3) They should have similar formal education (all three hold a DMA or PhD, and all studied at universities in *Instituto de Artes* – UNESP).
(4) They should have experience in musical performance.
(5) They should have previously participated in juries for sight-singing and music performance.
(6) As tenured professors, they should have gone through stringent examinations before receiving their jobs and should have had at least 3 years of work to show that they can maintain professional status, according to Brazil’s country laws.
(7) They should receive the same assessment instructions.

The judges should not be singers; singers tend to be too demanding concerning vocal technique, and that should not be the case since students are not all specialized in that matter, although they are all musicians (among them, many are instrumentalists, not singers). We believed, from experience, that having a singer in the jury would affect the evaluations negatively. For instance, when judging intonation, in this context, it was important (and that was made explicit to the judges) to recognize the pitches and melodies, but pitches were not expected to be as accurate as if they were sung by a professional singer. In addition, the great majority of teachers of musical perception are not singers; nevertheless, they are experts in teaching and perceive tuning in diverse musical contexts. The three judges are or have been teachers of musical perception. On the other hand, in a singing contest, we believe it is mandatory that all judges are professional singers.

Although we considered it important to take into consideration the professional background of the judges in terms of their recognition by peers (since they all have gone through stringent examinations as professors), and their experience in music performance and teaching, we do not attach importance to the kind of instrument they play.

### Materials

The theoretical model of the tonal sight-reading trait that we applied in the assessment of the 10 melodies consisted of four criteria: (a) intonation and pitch accuracy; (b) tonal sense and memory; (c) rhythmic and metric precision; and (d) fluency and direction. This model has been used for 6 years as part of evaluation rubrics in the Music Department of the *Instituto de Artes* at UNESP. We developed these criteria during our years of practice regarding student evaluation, and they have been neither validated nor tested in terms of construct validity and judges’ agreement. Because agreement among judges is a fundamental piece of information in the process of developing a tool, this was the main question we asked in this study: Do experts agree on the given criteria for scoring?

The criteria are in a 5-point Likert format (i.e., an ordinal measurement with four thresholds); the lowest score indicates low ability, with a melody that was not recognizable, and the highest score refers to a melody that could be fully recognized in each of the four aspects (see section “Materials and Methods”).

Our tool was developed in a context with specific criteria used to assess various components of sight-singing. This strategy has been adopted in the majority of psychological scales; each criterion has various categories of answers and a description of how good or bad a given participant is for that criterion. Researchers have discussed whether it would be better to have a general measure or a protocol with specific items (Stanley et al., 2002). We opted to develop a tool consisting of four criteria (items) because this would allow future researchers to explore construct validity via confirmatory factor analysis (Bollen, 1989), thus investigating the psychometric features of our criteria and the amount of common variance among them.

We expected that the *fluency and direction* criterion would be unlikely to have high values of agreement because the judges could have different perspectives and theoretical approaches. However, we believed that the other three criteria would not pose any difficulty, as they have been traditionally taken for granted in Western music performance assessment, particularly the item about *rhythmic and metric precision*. Each criterion’s difficulty levels and discrimination can be better evaluated via factor analysis, which is not the purpose of the current study.

Each judge independently filled out a spreadsheet containing the four criteria using a 5-point Likert scale (scores of A, B, C, D, or E; see Appendix C for complete descriptions of each category) for each melody sung (see Appendix A).

For the first criterion (*intonation and pitch accuracy*), we instructed the judges to recognize the pitches of the melody sung, from the lowest to the highest level. We gave the same kind of instruction for the other criteria, with variations according to each quality. For instance, on the second criterion (*tonal sense and memory*), we instructed the judges to evaluate the students’ capacity to maintain a consistent tonic (or modulating tonality, if there was one). Although this seems to be correlated to the first item (*intonation and pitch accuracy*), we conceived it to address a different kind of memory; for instance, an individual could make pitch errors but still be capable of recovering by remembering central pitches.

#### Judgment of Sight-Singing Skills of Intervals

The three judges used a 3-point Likert scale (*incorrect*, *out of tune*, or *correct*) to assess the participants’ sight-singing skills across 19 melodic intervals (see Appendix B). The judges independently listened to and scored the 50 recordings; participants’ names were blinded during the scoring procedures, and the judges had access to participants’ numeric IDs only.

#### Sample Size and Statistical Analysis

The input parameters used to estimate the number of students being evaluated were power (80%), level of significance (5%), a κ less than the null-hypothesis value of 0.2 (i.e., a slight value), and a κ less than the alternative hypothesis of 0.58 (i.e., a fair-agreement value); these values were selected because, though we did not expect high values of agreement, we did expect to reach at least the null-hypothesis value of 0.2. These input parameters indicated a sample size of 50 participants. In terms of number of raters, we opted for three, *a priori*, because, according to Shoukri et al. (2004), when seeking to detect a κ of 0.40 or greater, it is not advantageous to use more than three raters per participant. Shoukri et al. (2004) argued that, for a fixed number of observations, increasing the number of raters beyond three has little effect on the power of the hypothesis tests or on the width of the confidence intervals. Consequently, Shoukri et al. (2004) showed that increasing the number of participants is a more effective strategy for maximizing power.

We conducted an analysis of the agreement among judges using weighted κ, following the guidelines described in Kottner et al. (2011) for the use of categorical and ordinal scales. The following cutoffs were used to evaluate the magnitude of the agreement, based on Zegers et al. (2010), who classified a κ value between 0.00 and 0.20 as *slight*; between 0.21 and 0.40 as *fair*; between 0.41 and 0.60 as *moderate*; between 0.61 and 0.80 as *substantial*; and between 0.81 and 1.00 as *almost perfect*. Negative values might occur; these would indicate that the observed probability of disagreement is larger than what would be expected by chance (Vanbelle, 2016). We ran all the analyses using STATA version 14 (StataCorp LP, 2013a) and an adopted significance level of 0.05. We declared some *p*-values as “not applicable, NA” because not all judges endorsed all the categories of answers for a given item. For example, one judge might have used A, B, and E for students but not used the C or D categories. This asymmetry in the prevalence of the answers, with some judge not using all the categories, led to non-computable *p*-values. Because of this situation, we calculated only point estimates of agreement. In other words, kappa can be calculated, but there is no test statistic for testing against κ > 0. If the number of ratings per subject vary, kappa only suppresses the calculation of test statistics. Therefore, the degree of agreement is interpretable. We report the *p*-values as NA in order to call the readers’ attention to such situations in which the judges are using a different number of items to evaluate the students’ performance on the tasks. The judges are using a number that is different from the predetermined number of five, not always endorsing the five categories for those items marked as NA. For example, judge A might have used only three of five categories of answers.

## Results

Supplementary Table 1, which provides details and a weighted combination for each answer category, shows the agreement among judges for the melodic sight-singing tasks across the 10 melodies. The columns named A, B, C, D, and E give information about how well the three judges agreed for each of the five answer categories. For instance, the category of the answer A column is the two-rating kappa, where positive is Category A and negative is Category B, C, D, and E. The test statistic, however, is calculated differently (see StataCorp LP, 2013b). The combined kappa, the sixth column, is the appropriate weighted average of the individual kappa; it is a general weighted measure that has taken into account the five answer categories.

For example, item 1 in melody 1, considering answer A as positive and all other answers as negative (answer B, C, D, and E), κ = 0.538 (*p*-value <0.001). For answer B in the same context (e.g., criterion and melody) it was observed that κ = 0.1319 (*p*-value = 0.053). Combining a weighted kappa considering the five estimated kappa for such items in melody 1 was κ _weighted_ = 0.353 (*p*-value <0.001).

It is important to understand the distinction between each answer category and the weighted composite of the agreement, not only in terms of general agreement (i.e., an overall reliability score for each criteria across the various melodies and intervals under evaluation), but also within each item. Thus, Supplementary Table 1 points out the areas in which the judges agree more (or less). *Tonal sense and memory* (Item 2) only reached a moderate level of Combined agreement among the judges in 7 out 10 melodies. For *rhythmic precision* and *regularity of pulse and subdivisions* (Item 3), the judges agreed moderately for only one of the melodies (the second). For *intonation and pitch accuracy* (Item 1) and *fluency and direction* (Item 4), the indices were either fair or slight for all melodies. Via visual inspection, we might note that the levels of agreement are not correlated with the complexity—be it rhythmically, melodically or harmonically—of the melodies. We also calculated an index of agreement for each outcome within each item, as shown in the columns labeled A to E. It should be noted that there is an extraordinarily large number of “not applicable,” which are *p*-values that are not computable in such situations. As mentioned in the Statistical Analysis section, this is due to some of the answer categories not being used by at least one judge.

As a simple way to summarize the ten obtained weighted kappa (κ_w_) for the four items, the mean for the κ_w_ was as follows: κ1_w_ = 0.296, κ2_w_ = 0.487, κ3_w_ = 0.224, and κ4_w_ = 0.244, ranging then from fair to moderate.

Supplementary Table 2 shows the agreement for interval sight-singing tasks. The obtained indices are better than those achieved in melodic sight-singing, for which the lowest Combined agreement (κ) was 0.406 and the highest was 0.792 (on average, κ = 0.634). For each item, we also calculated an index of agreement for the outcomes of the *correct*, *out of tune*, and *incorrect* answers.

## Discussion

We expected that the last item, *fluency and direction* (Item 4), would obtain poor indices of agreement (the highest combined kappa found was 0.41, a fair agreement) even after experts had received detailed instructions and evaluation criteria, as the theoretical background (e.g., Lester, 1986) was not familiar to all judges. In addition, this item raises complex music questions that can create doubts and biases; it should, perhaps, be presented in separate items, such as *fluency in terms of interruptions* in one item and *desirable or unwanted accents* in another. However, contrary to what many instrument and voice professionals or teachers are used to thinking, manifested variable items such as rhythmic precision and intonation—some of the most-used criteria in music performance assessment, whether vocal or instrumental—are not very easy to agree upon. *Intonation and pitch accuracy* (Item 1) reached the maximum of 0.403 combined kappa (a fair agreement), and *rhythmic precision, regularity of pulse and subdivisions* (Item 3) reached mostly slight or fair results (from 0.162 to 0.230), and a moderate level of 0.458 only in Melody 2. Note that Latimer et al. (2010) and Norris and Borst (2007) found the same results for the rhythmic dimension. It is also possible that *pitch accuracy and intonation* should be separated into different items as well, as *metric* and *rhythmic precision* *at subdivision levels*, with both of them being gradable in more general terms, such as from lowest achievement to highest achievement.

Wrigley and Emmerson (2011, p. 115) argued that “it has to be recognized that the aesthetic nature of performance assessment is likely to always retain some component of indefinable subjectivity,” which “will remain inaccessible through the use of a verbal and numerical rating scale.” However, we refuse to infer that the results indicating poor agreement in melodic sight-singing are due to subjectivity in the assessed construct; in fact, we believe that it is fundamentally imperative that the areas of music perception and music performance develop and test their tools to create criteria for public competitions regarding jobs, solo contests, music schools, conservatoires, and universities. Moreover, criteria are fundamental to the establishment of standardized evaluations. In terms of academic-achievement evaluation, scholars in the psychology and education areas have been successful in using robust statistical inferences (e.g., item-response theory and other techniques derived from structural equation modeling) since the 1970s to create and validate tools for assessing cognitive processes as subjective as music performance seems to be. It is interesting that the assessment of music performance, especially at higher levels of expertise, has been so poorly tested and that no defined criteria or standardized tools have been used. In this study, we have shown that items that are considered efficient or even taken for granted as common sense by professional musicians, nonetheless do not reach good levels of agreement, which is a fundamental feature of any criteria.

Low κ values indicate that the investigated measure or classification instrument is unable to make clear distinctions between the subjects of the population when those distinctions are very rare or difficult to achieve (Kraemer et al., 2002; Vach, 2005). In addition, this might reflect the raters’ inability to distinguish between adjacent categories (Darroch and McCloud, 1986).

Furthermore, Supplementary Table 1 indicates that a smaller number of options could have worked better than the five given alternatives; however, due to the nature of the assessment in terms of complexity when comparing interval and melodic tasks, it is difficult to dissociate the effects regarding complexity from the availability of outcomes that the judges are choosing. Columns B through D show mostly fair to slight indices of agreement, but column A (lower ability) shows better levels of agreement (the highest level of agreement of 0. 916 was found in Melody 7 at Column A, Item 1). Interestingly, although column E, which refers to higher ability, shows better levels of agreement than the internal columns do, it only presents excellent levels for the second item (*tonal sense and memory*): highest level of 0.832 appears in Melody 1. It seems that good levels of agreement are more frequently reached for lower levels of expertise than for higher levels.

Qualitatively speaking, one of the judges commented that it was very hard to make decisions in terms of the detailed information provided, especially for the first item (*pitch accuracy and intonation*). He said it was difficult to reason the precise amount of diversion from correct pitches (e.g., more than a half step raised or dropped, less than a half step raised or dropped) performed by students. It seems that it would be more effective to establish the scale in more general terms, gradable from lowest achievement to higher achievement, even considering specialists as judges, since both tasks (singing and evaluation) are performed very rapidly.

We recognize that one of the limitations of this study concerns the fact that training failed to provide a thorough understanding of theoretical support for the fourth item (*fluency and music direction*).

In terms of choice, according to Schwartz (2007a), as the number of options increases, the effort required to make a good decision escalates as well, which is one of the reasons that choice can be transformed from a blessing into a burden. It is also one of the reasons that we don’t always manage the decision-making task effectively. (p. 48)

Schwartz (2007b, p. 50) added that “choice overload can paralyze people into indecision, heighten regret, and decrease satisfaction with even good decisions.” Arunachalam et al. (2009) argued that what they call excessive-choice effect is less prevalent than what a previous study by Schwartz et al. (2002) suggested, although the former focused on individual preferences in terms of affecting consumer behavior, and the latter focused on the level of satisfaction gained from a choice made. For Arunachalam et al. (2009, p. 824), “further studies on how to help firms establish a large product variety without discouraging consumers” is a concern. For Schwartz et al. (2002, p. 1195), “it is critical for future research to clarify whether being maximizers makes people unhappy or being unhappy makes people maximizers.”

For each interval, the judges had only three options to categorize the student (*correct*, *out of tune*, and *incorrect*). Because there were fewer categories than the five used in melodic sight-singing tasks, and because only two notes were sung, the judges were more restricted in terms of possibilities and, therefore, their level of agreement *per se* may have increased due to the way the judges obtained information (see the “Combined” column of Supplementary Table 2: Interval 13–an ascendant perfect fourth–reached the highest level of agreement of 0.792, while the lowest level of 0.406 is found at Interval 11–a major descendant seventh). Nevertheless, it is important to point out that the nature of the tasks being evaluated requires the participants and judges to have different skills.

In the “Out of tune” column in Supplementary Table 2, the indices of agreement achieved moderate to slight values (from 0.479 to 0.004), but the “Correct” and “Incorrect” columns reached mostly moderate to excellent values (0.348–0.925). Although the present study is not concerned with the degree of happiness or with encouraging consumers, our results do show that bias decreases with fewer alternatives as the degree of agreement increases. In other words, even when a judge has only three alternatives to evaluate the performance of two pitches, it would be better for the judge to have only dichotomous items.

A limitation of this study concerns the order of listening and rating, which was determined by the judges, regardless of the blind evaluation. Glejser and Heyndels (2001) showed that musicians who performed later in a given contest obtain better scores, on average.

Future research using structural equation modeling (such as confirmatory factor analysis and item-response theory) might reveal how harsh and discriminative the set of items are for both tasks, in parallel with the construct validity of both tasks. Confirmatory factor analysis is an indispensable analytic tool for construct validation (also called factorial validity or internal consistency), although it has rarely been applied to the area of music performance.

## Conclusion

Although this study addressed sight-singing assessments, due to similarities in evaluation items of performance, these findings lead to a discussion on the disagreement and validity of models that have been used in assessing high-level music performance, raising issues on the need to create trustworthy items and evaluation criteria in music auditions and contests. It seems that complexities of music making challenge ecological evaluation in that it was difficult for the judges, for example, to separate sound quality from intonation. In other words, once they enjoyed the color of a voice, they tended to minimize minor variations in pitch intonation. This valuable information points to the weight those qualities of performance are given by listeners in judging it, and that can help to better understand and build a model for future research that might be closer to real performance and reception constituted by observable variables.

Thompson and Williamon (2003, p. 38) mention that it might be “actually irrelevant whether an evaluator arrives at their final quality judgment through the weighing-up of purely ‘musical’ factors or because they take exception to a particular item of clothing worn by the performer,” but for those who are auditioning, such reasons might not be irrelevant. Although it is true that “simply using the same protocols that are prevalent in educational contexts may not provide them with the degrees of reliability, replicability, and discrimination demanded by scientific standards” (Thompson and Williamon, 2003, p. 38), it is also true that many lives that have been affected by bias can, once the evaluation criteria are clarified, go in a different direction. If, for instance, visual aspects of performance turn out to be an important issue (as might the case for opera singers or soloists), said factors are certainly not crucial in instrumental auditions for an orchestra of an opera house. On the other hand, if visual aspects are considered crucial, they should be included as items to be evaluated, and they also should be detailed (i.e., they should imply assertiveness and apparent comfort, both in the task and in the clothes). We believe that, without validated tools to evaluate music at higher levels of performance, further studies will have to be conducted to address this issue.

This study was purposefully conducted utilizing audio-recorded performances only in order to deny visual biases. Since visual aspects of performance, as stated in Platz and Kopiez (2012) and Tsay (2013), are important in the reception of music, researchers in further studies should consider evaluating items (criteria) that address such aspects as well.

## Author Contributions

GB and NG contributed with conception and design of the study. HC-M performed the statistical analysis. GB wrote the first draft of the manuscript. NG wrote sections of the manuscript. All authors contributed to manuscript revision, read, and approved the submitted version.

## Conflict of Interest Statement

The authors declare that the research was conducted in the absence of any commercial or financial relationships that could be construed as a potential conflict of interest.
