# Supplementary figures and images for: (Dis)agreement on Sight-Singing Assessment of Undergraduate Musicians
Source: Front Psychol. 2018 May 29;9:837. doi: 10.3389/fpsyg.2018.00837 (PMC5987045; doi:10.3389/fpsyg.2018.00837)

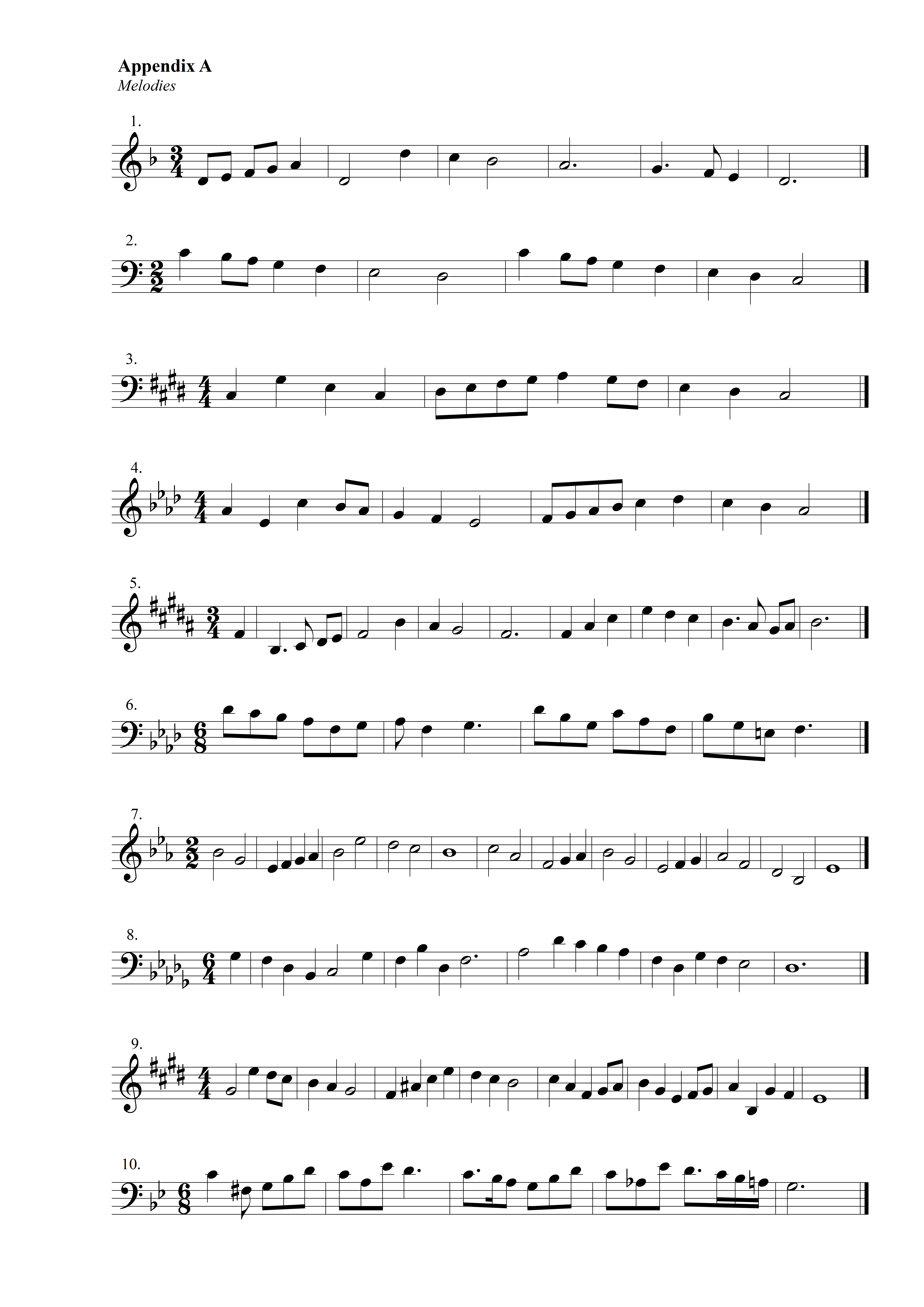

Supplement: Supplementary file 3 [file Image_1.TIF]

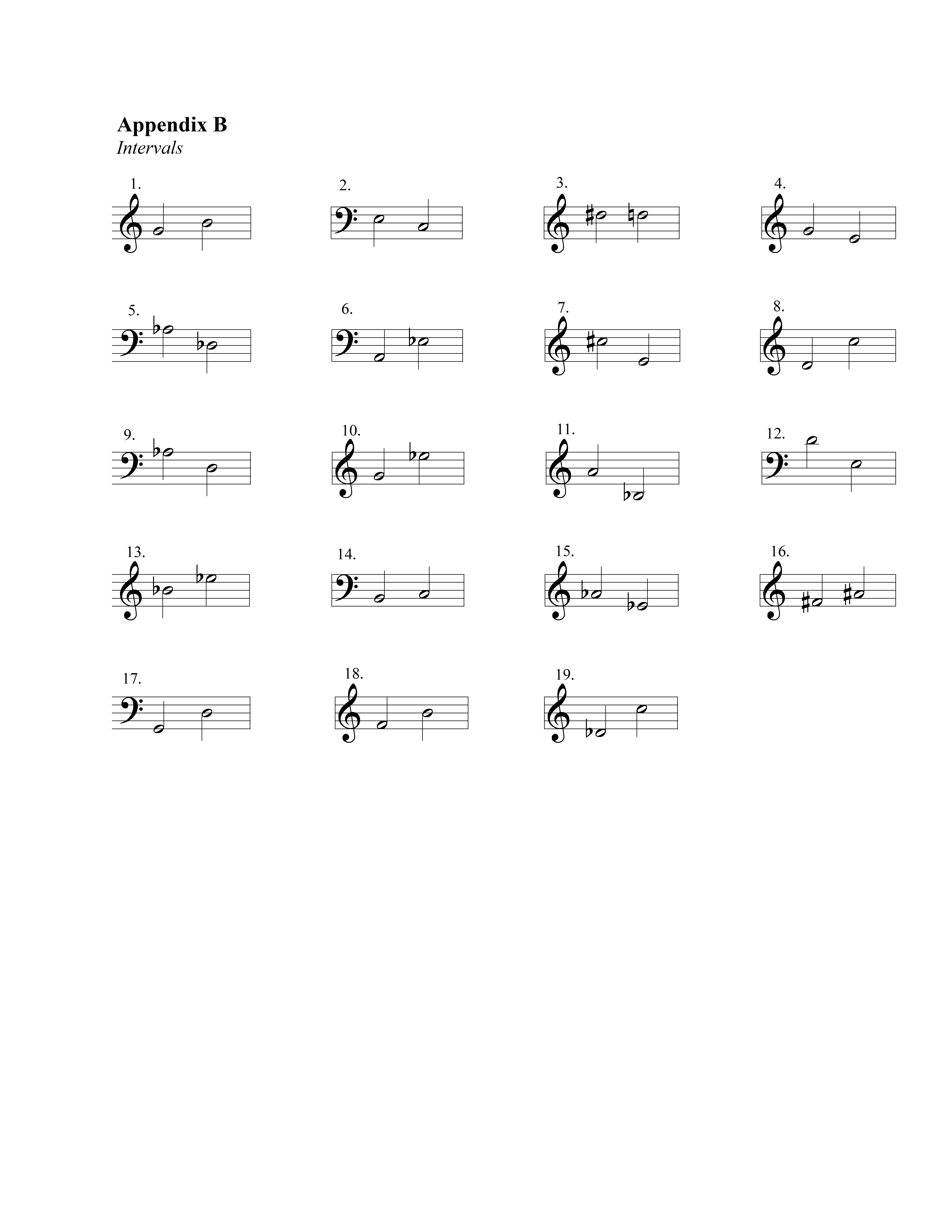

Supplement: Supplementary file 4 [file Image_2.TIF]
