# Supplementary material for: (Dis)agreement on Sight-Singing Assessment of Undergraduate Musicians
Source: Front Psychol. 2018 May 29;9:837. doi: 10.3389/fpsyg.2018.00837 (PMC5987045; doi:10.3389/fpsyg.2018.00837)
Supplement: Supplementary file 5 [file Image_3.PDF]

Appendix C

Criteria description

1. **Intonation and pitch accuracy**: at this item, one should not consider the kind of precision expected as if singers were being judged by a specialist jury. Instead, it should be considered that students from diverse backgrounds in music, such as instrumentalists, conductors, composers, or music educators are being evaluated. In other words, the degree of vocal technique expected is not that of a singer, but one should be able to recognize the pitches of the melody sung. For a better understanding, consider, for the columns A to E, the following order for this item:
   A. It is not possible to recognize the melody sung at all.
   B. It is possible to recognize the melody, but the intonation drops (or raises) at least a half step.
   C. It is possible to recognize the melody, but the intonation drops (or raises) up to a half step.
   D. It is possible to recognize the melody, but the intonation drops (or raises) less than a half step.
   E. It is possible to recognize the melody fully.

2. **Tonal sense and memory:** although related to item 1 ("intonation and pitch accuracy"), it was conceived to approach a different kind of memory, for instance, when an individual gets lost in terms of pitch during the solfege of a tonal or modal melody, or makes errors of decoding pitches, but is still capable of recovering through tonal memory or pitches just sung. Hence, for this item, the judge should consider the following classifications for the columns A to E:
   A. It is not possible to recognize the tonality(ies) sung at all.
   B. It is possible to recognize the tonic (and eventually the dominant) of the tonality(ies) only.
   C. It is possible to recognize the tonality(ies), but occasional errors of tones occur.
   D. It is possible to recognize the tonality(ies) and diatonic tones, but chromaticism (chromatic inflexions of diatonic tones) are not realized.
   E. It is possible to recognize the tonality(ies) fully, even in case where subtle instabilities of intonation occur during the solfege.

3. **Rhythmic precision, regularity of pulse and subdivisions:** understood here as the capacity of participants on decoding rhythms, meters, and on maintaining pulse and subdivisions regularity during all the exercise. It is not expected, naturally, that the metronomic speed be maintained during the whole solfege, but, even though changes of speed at the pulse level may occur, they are supported by changes at the subdivision levels. Actually, such a thing is quite desirable, musically, since it denotes control and experience by the participant (on doing *ritardandos* at the end of a phrase, for instance). Hence, to evaluate the performance of the participant on that item, the judge should consider the following choices for the columns A to E:

A. It is not possible, absolutely, to discern both meter(s) and regularity of pulses and subdivisions.
B. It is possible to discern meter(s), but subdivisions are inconsistent (for example, a participant executes a ternary subdivision instead of a given binary one, and vice-versa).
C. It is possible to discern meter(s) and its(their) hierarchical subdivisions, but many inconsistences (errors) occur in the course of the solfege.
D. It is possible to discern meter(s) and its(their) hierarchical subdivisions, but occasional inconsistences occur in the course of the solfege.
E. It is perfectly possible to discern meter(s) and its(their) hierarchical subdivisions, and, when there are flotations of speed, they are supported proportionally by the hierarchical levels of division and subdivisions of the beat (*rubato, rit.*).

4. **Fluency and music direction:** refers not only to the capacity of the participant on moving forward regardless of mistakes, but also on doing so (with or without mistakes) in a way that the musical discourse is clearly expressed. The expression *musical discourse* should be understood here by the capacity of the student to prevent inadequate accentuations[1], being them related to meter, rhythm, or harmony[2], that prevent the fully expression of musical prosody[3]. Hence, to evaluate the performance of the participant on that item, the judge should consider the following choices for the columns A to E:
   A. It is not possible to follow the musical discourse.
   B. It is possible to follow the musical discourse, but many interruptions occur.
   C. It is possible to follow the musical discourse, but tiny non-intentional delays occur (a brief non-desired fermata, for example, while the student ‘takes time’ in order to decode pitches and rhythms).
   D. It is possible to follow the musical discourse, but extra-accents occur that prevent its total fluency.
   E. It is possible to follow the musical discourse fully.

---

[1] Inherent accentuations of the musical discourse (in tonal music): “the accentuation on a long note is quality inherent in the note, not one added on the volition of the performer” (LESTER, 1986, p. 266). Hence, technical questions, for instance, or doubts about which notes are hierarchically more important (goals, as opposed to ornamentation or passage notes, for example), that end up causing non-intentional accents, are not considered inherent in the musical discourse.
[2] One should consider, here, harmony perception as it is implied in the musical discourse.
[3] Musical prosody is taken metaphorically here, in relation to musical accents, since it doesn’t refer to words, but exclusively to music.
